# An Open‐Label Study to Assess the Efficacy and Tolerability of a Multifunctional, 10‐Peptide Face and Neck Serum to Address Skin Quality

**DOI:** 10.1111/jocd.70746

**Published:** 2026-03-09

**Authors:** Kimberly Cayce, Kristen M. Jacobs, Savanna Boda

**Affiliations:** ^1^ Cayce Dermatology Center & Medical Spa Columbia Missouri USA; ^2^ Ooh La La Spa, Anti‐Aging & Wellness Glen Carbon Illinois USA; ^3^ Savanna Boda Aesthetics Southlake Texas USA

**Keywords:** facial skin rejuvenation, neck skin rejuvenation, peptide serum, skin quality

## Abstract

**Background:**

There is increasing patient demand for improved skin quality and the desire to achieve rejuvenated skin and improve self‐confidence.

**Aims:**

The objective of this 12‐week study was to assess changes in skin quality and subject satisfaction after using a novel anti‐aging 10‐peptide serum (Pep Up Collagen Boost Face & Neck Serum; Colorescience Inc., Carlsbad, CA).

**Patients/Methods:**

Healthy adult subjects seeking facial and neck skin rejuvenation (*N* = 48) were divided into two groups. Group A included subjects not currently using prescription, medical grade, advanced, or physician‐dispensed skincare products and Group B included subjects who currently were using such products. Subjects in both groups applied the anti‐aging peptide serum twice daily as part of their daily skincare routine for 12 weeks and were evaluated after 4, 8, and 12 weeks of treatment. Improvements in Global Skin condition and other parameters associated with skin quality were assessed by three investigators.

**Results:**

By Week 12, 89.6% of subjects showed clinical improvement (*p* < 0.01), with most (52%) achieving moderate‐to‐marked global improvement. Both groups had median global scores of 3.0, with similar mean scores (Group A, 2.6; Group B, 2.8). Subjects in both groups indicated improvements in global scores (Group A, 91.7%; Group B, 87.5%) and most subjects in both groups demonstrated marked improvements from baseline (54.2% and 50.0%). The product was well‐tolerated and subject satisfaction was high.

**Conclusions:**

The twice‐daily application of a novel anti‐aging 10‐peptide serum restores several markers of facial and neck skin quality that were incremental to basic skincare routines and professional skincare regimens.

## 1 Introduction

The field of cosmetic dermatology has long focused on improving age‐related changes in facial appearance by tightening the skin to reduce fine lines and wrinkles, restoring tissue volume, and reducing hyperpigmentation. This has been accomplished using a variety of treatment strategies including lasers, fillers, peels, microneedling, and neurotoxins [1, 2, 3, 4]. While these remain valuable tools for those seeking facial rejuvenation, patients continue to seek topical products to enhance skin quality as a significant component of facial attractiveness. Markers of skin quality include even skin tone, skin hydration, skin firmness, skin elasticity, surface smoothness, and skin glow or radiance [5]. It is well recognized that skin quality can have a substantial impact on one's emotional health, quality of life, self‐perception, and interpersonal relations with others [6, 7]. Skin quality is an important component of human attractiveness [5], projecting overall health and well‐being [8] and even perceived character traits [9]. It has also become apparent that numerous environmental insults can have a significant negative impact on skin quality including tobacco smoke [10, 11], ultraviolet light [12, 13], high‐energy visible light [14], and air pollution [15, 16, 17].

A novel anti‐aging peptide serum has been formulated with 10 peptides, antioxidants, botanicals, and other ingredients which hydrate the skin, improve skin tone, texture, and firmness, and reduce fine lines and wrinkles while protecting against environmental insults (Pep Up Collagen Boost Face & Neck Serum; Colorescience Inc.) (Table 1). Decaplex‐10, a patented blend of 10 different peptides, provides a comprehensive approach to rebuilding collagen and elastin in the skin. These peptides include a combination of signaling and inhibiting peptides that build collagen and elastin, prevent visible signs of aging, and enhance skin quality. For example, hexapeptide‐2 is an enzyme‐inhibitor that visibly brightens skin by decreasing the appearance of dark spots on the skin. Acetyl tetrapeptide‐9 is a signaling peptide that visibly improves skin thickness and firmness for an overall firming effect. Copper tripeptide‐1 is a signaling and carrier peptide that stimulates collagen and elastin production to make the skin feel more supple and look visibly smoother. A complete list of the 10 peptides in Decaplex‐10 is provided in Table 2. Botanical ingredients include *Pseudoalteromonas* sp. with beneficial effects on melanogenesis [27], *Physalis angulata* and *Glycine soja* with anti‐inflammatory activity [28, 29], *Tremella fuciformis* which inhibits ultraviolet photodamage of the skin [30, 31], and *Avena sativa* which has antioxidant properties and restores skin barrier function [32, 33]. Additional ingredients such as antioxidants and those that provide deep hydration are known to optimize the performance of skin peptides [34].

**TABLE 1:** Study product ingredients.^a^

Water, isoamyl laurate, glycerin, polyacrylate‐13, mannitol, butylene glycol, polyisobutene, *Pseudoalteromonas ferment* extract, caprylyl glycol, *Physalis angulata* extract, sodium hyaluronate, caprylic/capric triglyceride, polysorbate 20, hydrolyzed wheat protein, sorbitan isostearate, hydrolyzed soy protein, ethylhexylglycerin, betaine, dimethylmethoxy chromanol, carbomer, palmitoyl tripeptide‐5, sodium lactate, xanthum gum, dipeptide diaminobutyroyl benzylamide diacetate, lecithin, *Tremella fuciformis sporocarp* (mushroom) extract, *Glycine soja* (soybean) oil, sodium oleate, disodium ETDA, hydrogenated lecithin, beta glucan, copper tripeptide‐1, tripeptide‐10 citrulline, tocopherol, *Avena sativa* (oat) kernel extract, acetyl tetrapeptide‐9, tripeptide‐1, palmitoyl tripeptide‐1, palmitoyl tetrapeptide‐7, hexapeptide‐2, sh‐oligopeptide1, triethanolamine, phenoxyethanol, caprylhydroxamic acid, potassium sorbate

^a^ Pep Up Collagen Boost Face & Neck Serum; Colorescience Inc.

**TABLE 2:** Decaplex‐10 peptide ingredients and functions.

	Peptide type	Function
Down‐regulating peptides		
Dipeptide diaminobutyroyl benzylamide diacetate	Neurotransmitter‐inhibitor	Reduces the appearance of dynamic wrinkles of expression [18]
Hexapeptide 2	Enzyme‐inhibitor	Visibly brighten skin by decreasing the appearance of dark spots on the skin [19]
Up‐regulating peptides		
Copper tripeptide 1	Signaling and carrier	Makes skin feel more supple and look visibly smoother by stimulating collagen and elastin production [20]
Palmitoyl tripeptide 1	Signaling	Provides synergy of two matrikines,^a^ Pal‐GHK and Pal‐GQPR, that support production of collagen and hyaluronic acid to reduce fine lines and wrinkles
Palmitoyl tetrapeptide 7		
Acetyl tetrapeptide 9	Signaling	Visibly improves skin thickness and firmness for an overall firming effect [21, 22]
Sh‐oligopeptide	Mimicking	A 53‐amino acid peptide with a chemical structure similar to epidermal growth factor which visibly renews the skin and increases skin firmness and tone [23]
Simultaneous up‐ and down‐regulating		
Palmitoyl tripeptide 5	Mimicking	Unique peptide offering a synergistic benefit to assist the body in its production of collagen [24]
Tripeptide‐10 citrulline	Signaling and carrier	Combination of peptides and proteins that help support the production of collagen [22, 25]
Tripeptide‐1 (also contains *Pseudoalteromonas* ferment extract and hydrolyzed wheat and soy protein)	Expression and glycation	

^a^ Matrikines are bioactive fragments released from extracellular matrix proteins which regulate several physiological processes [26].

It has been shown that when skin quality improves, people report feeling less self‐conscious and more self‐confident [35, 36]. Consequently, there is increasing patient demand for improved skin quality and the desire to achieve healthy skin [7]. Therefore, the objective of this study was to assess changes in a battery of skin quality markers following the twice‐daily use of the anti‐aging 10‐peptide serum in subjects currently using daily over‐the‐counter and professional skincare treatments. These include some subjects using topical tretinoin and retinol, which are known to improve sun damaged and aging skin [37]. Per inclusion criteria, subjects using professional grade skincare treatments were required to have used these products for a minimum of 90 days prior to the study initiation and to maintain the same dose and frequency throughout the study. The study also assessed the safety and tolerability of the study product.

## 2 Materials and Methods

### 2.1 Study Subjects

Enrolled subjects were healthy adults, 18 to 75 years old, of any race or ethnicity, and any Fitzpatrick skin type who were seeking treatment for mild‐to‐severe skin quality concerns of the face and neck. Enrolled subjects expressed their willingness to follow all study requirements including avoiding first time lash extensions or facial tattoos and discontinuing the use of any home facial treatment devices during the 12‐week study.

Reasons for exclusion from study participation included treatment during the previous 3 months with neuromodulators, dermal fillers, chemical peels, microneedling or energy‐based devices, such as intense pulse light, broadband light, fractional resurfacing, CO_2_ resurfacing, ultrasound therapy, microneedling with radiofrequency, and other similar device treatments, or facial cosmetic or reconstructive surgery during the previous 6 months. Subjects who were pregnant, planning to become pregnant, or were nursing were not allowed to participate. Subjects agreed to maintain all exclusion criteria and avoid sun exposure without applying sunscreen as directed by a study Investigator.

Subjects were divided equally into two treatment groups. Subjects in Group A had previously or currently used only over‐the‐counter skincare products but no professional‐grade skincare products. Subjects in Group B were using professional‐grade skincare products for a minimum of 90 days (Table 3). These treatment groups represent the two types of patients that seek professional skincare recommendations—those who are naïve to professional skincare and those who receive professional skincare but wish to boost their results or overcome treatment plateaus.

**TABLE 3:** Professional‐grade product categories used by Group B used ≥ 90 days before and during the study.^a^

Cleanser types: with and without acids
Serums types: antioxidants, hyaluronic acid, peptides, lipids
Non‐hydroquinone skin lighteners
Moisturizer types: with and without lipids, antioxidants, peptides, or acids
Topical retinoid types: retinol and tretinoin
Acid‐based peel pads
Sunscreen type: mineral and chemical

^a^ Brand identifiers excluded to reduce potential commercial bias.

### 2.2 Investigational Product

The investigational product is formulated to promote healthy collagen and elastin production, treat dryness, dullness, sagging skin, and wrinkles, and protect against the damaging effects of pollution and other environmental aggressors (Table 1) (Pep Up Collagen Boost Face & Neck Serum; Colorescience Inc.).

### 2.3 Study Procedures

#### 2.3.1 Group A

Each morning, subjects randomized to Group A washed their faces with a cleanser provided by the study sponsor to use for the duration of the study. Immediately after cleansing, subjects applied two pumps of the study product to the face and one pump to the neck. This was followed by an application of a sponsor‐provided SPF‐50 sunscreen lotion and an SPF‐50 brush‐on sunscreen for reapplication to the face and neck at least three times throughout the day. Each evening, subjects washed their faces with the sponsor‐provided cleanser. Immediately after cleansing, subjects applied two pumps of the study product to the face and one pump to the neck.

#### 2.3.2 Group B

Each morning, subjects randomized to Group B washed their faces with their current professional‐grade cleanser. Immediately after cleansing, subjects applied any prescription products, such as professional‐grade serums, as instructed by their skin health professional and applied two pumps of study product to the face and one pump to the neck. In order of viscosity (lightest to heaviest), subjects applied any other professional‐grade moisturizers followed by the application of their professional‐grade sunscreen and reapplied their sunscreen to the face and neck at least three times throughout the day. Each evening, subjects washed their faces with their current professional‐grade cleanser. Immediately after cleansing, subjects applied any prescription products, such as professional‐grade serums, as instructed by their skin health professional. They applied two pumps of study product to the face and one pump to the neck. They applied any other professional‐grade moisturizers in order of viscosity (lightest to heaviest).

### 2.4 Investigator Clinical Grading Assessments

Skin quality evaluations were conducted at Week 0 (baseline), Week 4, Week 8, and Week 12. To assist with assessments, digital imaging was obtained at Baseline and Weeks 4, 8 and 12 (VISIA Imaging System. Canfield Scientific; Parsippany, NJ). Images included standard capture, brown channel, red channel, wrinkles, texture, and pores for facial assessments and standard imaging for neck evaluation with three views at all time points.

For each subject, the investigator rated the following facial quality markers: Glow/Luminosity, Dullness, Pigmentation, Skin Tone, Pore Size, Acne Scarring, Redness, Dryness, Oiliness, Hydration, Fine Lines, Wrinkles, Laxity, and Crepiness. Neck quality markers were Redness, Hyperpigmentation, Uneven Skin Tone, Laxity, Crepiness, Rough/Uneven Texture, Fine Lines, and Wrinkles. The investigator rated facial skin and neck quality markers as None, 0; Mild, 1; Moderate, 2; and Severe, 3.

The Global Improvement Scale assessed overall facial aesthetic improvement at Weeks 4, 8, and 12. For each subject, the investigator rated changes in face and neck skin as 0, worse; 1, no improvement; 2, mild improvement (25% overall improvement); 3, moderate improvement (50% overall improvement); or 4, marked improvement (75% overall improvement).

### 2.5 Subject Study Assessments

Subjects completed digital questionnaires at each visit assessing baseline skin concerns, self‐perceived improvements in specific skin quality markers, overall skin appearance and confidence measures, treatment adherence and tolerability, quality of life impacts, willingness to continue use, and recommending the product to others.

### 2.6 Tolerability Assessments

The investigator assessed objective test product tolerability with an emphasis on erythema, edema, dryness, and scaling at Weeks 4, 8, and 12 (Table 4). Other adverse events were to be gathered as reported.

**TABLE 4:** Product tolerability assessments.

Assessment	Score
Erythema	0, No erythema of the treatment area. 1, Mild. Slight, but definite redness of the treatment area. 2, Moderate. Definite redness of the treatment area. 3, Severe. Marked redness of the treatment area
Edema	0, No edema/swelling of the treatment area. 1, Mild. Slight, but definite edema of the treatment area. 2, Moderate. Definite edema of the treatment area. 3, Severe. Marked edema of the treatment area
Dryness	0, No dryness of the treatment area. 1, Mild. Slight, but definite dryness of the treatment area. 2, Moderate. Definite dryness of the treatment area. 3, Severe. Marked dryness of the treatment area
Scaling	0, No scaling of the treatment area. 1, Mild. Barely perceptible, fine scales in limited areas of the treatment area. 2, Moderate. Fine scaling generalized to all areas of the treatment area. 3, Severe scaling and peeling of skin over all areas of the treatment area
Burning	0, No burning of the treatment area. 1, Mild. Slight burning sensation of the treatment area; not really bothersome. 2, Moderate. Definite warm, burning of the treatment area that is somewhat bothersome. 3, Severe. Hot burning sensation of the treatment area that causes definite discomfort and may interrupt daily activities or sleep
Stinging	0, No stinging of the treatment area. 1, Mild. Slight stinging sensation of the treatment area; not really bothersome. 2, Moderate. Definite stinging of the treatment area that is somewhat bothersome. 3, Severe. Marked stinging sensation of the treatment area that causes definite discomfort and may interrupt daily activities or sleep

### 2.7 Statistical Analysis

Descriptive statistics were calculated for all baseline characteristics and outcome measures. Continuous variables were presented as mean (SD) and categorical variables as frequencies and percentages. Between‐group comparisons at baseline used appropriate parametric or non‐parametric tests depending on data distribution. Longitudinal changes from baseline were analyzed using appropriate repeated measures ANOVA. The primary analysis population included all subjects who completed the study and had evaluable data, excluding those who withdrew or were lost to follow‐up (*n* = 8). All analyses were performed using R‐Project Statistical Software (https://www.r‐project.org/) with statistical significance set at *p* < 0.05. To determine the proportion of improved measures per subject, the percentage of baseline skin quality problems showing improvement at Week 12 was calculated. This involved identifying all measures where the subject had a baseline rating greater than zero, counting how many of these measures showed any decrease (improvement) in rating, then calculating the proportion.

### 2.8 Ethics

Each subject provided written informed consent prior to participating in any study‐related activity. This study protocol was approved by a commercial Institutional Review Board (WCG IRB, Puyallup, WA). This study conformed to Title 21 Code of Federal Regulations 50.25 and all U.S. laws and regulations governing Good Clinical Practice. Permission to reproduce digital images was obtained from each subject.

## 3 Results

### 3.1 Baseline Assessment

Forty‐eight subjects were enrolled into Group A (*n* = 24) and Group B (*n* = 24). The demographics and baseline characteristics of enrolled subjects are summarized in Table 5. There were equal numbers of male and female subjects in each group, with no significant between‐group differences in clinical characteristics. While 14.6% of subjects were not initially bothered by their skin appearance, the remainder reported being somewhat (58.3%), very (22.9%), or extremely self‐conscious (4.2%) about their skin appearance.

**TABLE 5:** Demographics and baseline characteristics.

	All (*N* = 48)	Group A (*n* = 24)	Group B (*n* = 24)
Mean age (SD), years	38.8 (13.5)	38.0 (12.6)	39.5 (14.6)
Gender, *n* (%)			
Female	38 (79.2)	19 (79.2)	19 (79.2)
Male	10 (20.8)	5 (20.8)	5 (20.8)
Fitzpatrick skin type, *n* (%)			
I	8 (16.7)	4 (16)	4 (16.7)
II	25 (52.1)	11 (45.8)	14 (58.3)
III	6 (12.5)	2 (8.3)	4 (16.7)
IV	5 (10.4)	5 (20.8)	0
V	2 (4.2)	2 (8.3)	0
VI	2 (4.2)	0	2 (8.3)

*Note:* Group comparisons were made using independent *t*‐tests for continuous variables and Fisher's exact test for categorical variables.

### 3.2 Investigator Global Improvement Score: All Subjects and Group Comparisons

Following the twice‐daily use of the investigational product, data analysis revealed progressive improvements in skin quality with a marked increase in moderate‐to‐marked improvements by Week 12.

**FIGURE 1:**
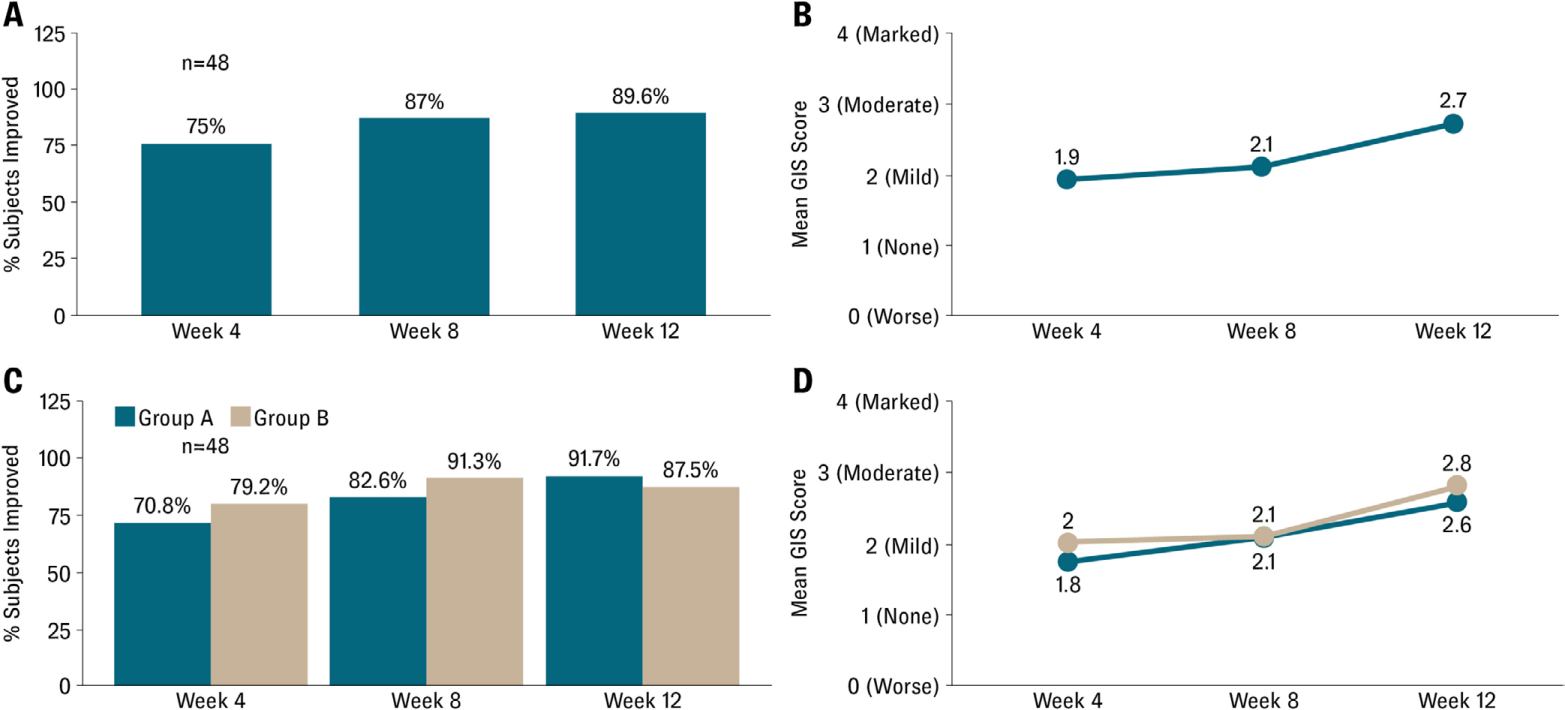
Clinical outcomes. Following 12 weeks of twice‐daily application of the test product, improvement in skin quality was achieved by 89.6% of subjects (*p* < 0.01) (A) and 52.1% achieved moderate‐to‐marked global improvement (B). Group A (*p* < 0.05) and Group B (*p* < 0.01) exhibited similar patterns of global improvement over the 12 weeks (C). Group B had a higher overall improvement rate, but Group A showed a higher proportion of marked improvement. At Week 12, both groups had similar mean quality scores (Group A, 2.6, Group B, 2.8) (D).

An analysis using a Friedman test confirmed a statistically significant improvement in Global Improvement Scores over time for the overall cohort (*p* < 0.01), Group A (*p* < 0.05), and Group B (*p* < 0.01), and Mann–Whitney *U* tests indicated comparable improvement patterns between treatment groups. At Week 12, 89.6% of subjects had improved, with 52.1% achieving moderate‐to‐marked global improvement (Figure 1A,B). A significant improvement was observed for the entire cohort (*p* < 0.01). At Week 4, most subjects were rated as having mild or moderate improvement (75%), while no subjects showed marked improvement. By Week 8, mild and moderate improvement reached 87%. A few were rated as worse (2.2%) or unchanged (10.9%); however, by Week 12, 29.2% of subjects were graded as having achieved marked improvement and 22.9% were graded as moderately improved.

Group A and Group B both exhibited similar patterns of global improvement over the 12‐week study (Figure 1C). At Week 8, while Group B had a higher overall improvement rate (91.3% vs. 82.6%), Group A showed a higher proportion of marked improvement (30.4% vs. 21.7%). At Week 12, both groups had a median score of 3.0, with similar mean quality scores (2.6 in Group A, 2.8 in Group B) (Figure 1D). The percentage rated as improved remained high and comparable between groups (Group A 91.7% vs. Group 87.5%), and the proportion of those with marked improvement was also similar (54.2% vs. 50.0%).

### 3.3 Investigator Ratings of Overall Facial Skin Quality Improvement

There was progressive improvement in the facial skin quality across all 12 parameters over the 12‐week study with lower mean ratings indicating reduced severity (Table 6). Notable improvements were seen in oiliness, decreasing from 1.33 at baseline to 0.33 at Weeks 8 and 12, and dryness, decreasing from 1.57 to 0.71 at Week 4 but increasing to 1.04 at Week 12. A significant improvement was also seen in wrinkles, decreasing from 1.56 at baseline to 1.08 by Week 8. Similarly, crepiness decreased from 2.09 to 1.36 at Weeks 8 and 12. These findings suggest subjects experienced measurable and sustained improvements in multiple markers of facial skin quality, particularly in texture‐ and hydration‐related concerns.

**TABLE 6:** Investigator ratings of improvements in facial skin quality markers.

Skin quality marker, *n* (%)	Baseline	Week 4	Week 8	Week 12	Significance
Laxity	1.86	1.68	1.59	1.64	*p* = NS
Crepiness	2.09	1.73	1.36	1.36	*p* < 0.01
Pore size	1.69	1.51	1.49	1.44	*p* = NS
Redness	1.66	1.38	1.32	1.36	*p* < 0.05
Hyperpigmentation	1.62	1.28	1.31	1.18	*p* < 0.01
Wrinkles	1.56	1.4	1.08	1.2	*p* < 0.05
Fine lines	1.59	1.24	1.1	1.27	*p* < 0.01
Roughness	1.38	1.24	1.15	1.0	*p* < 0.01
Dryness	1.57	0.71	0.83	1.04	*p* < 0.01
Acne scars	1.45	1.0	1.05	0.55	*p* < 0.01
Oiliness	1.33	0.9	0.33	0.33	*p* < 0.01

*Note:* Excludes subjects with a value of 0 at baseline.

Abbreviation: NS, not significant.

**FIGURE 2:**
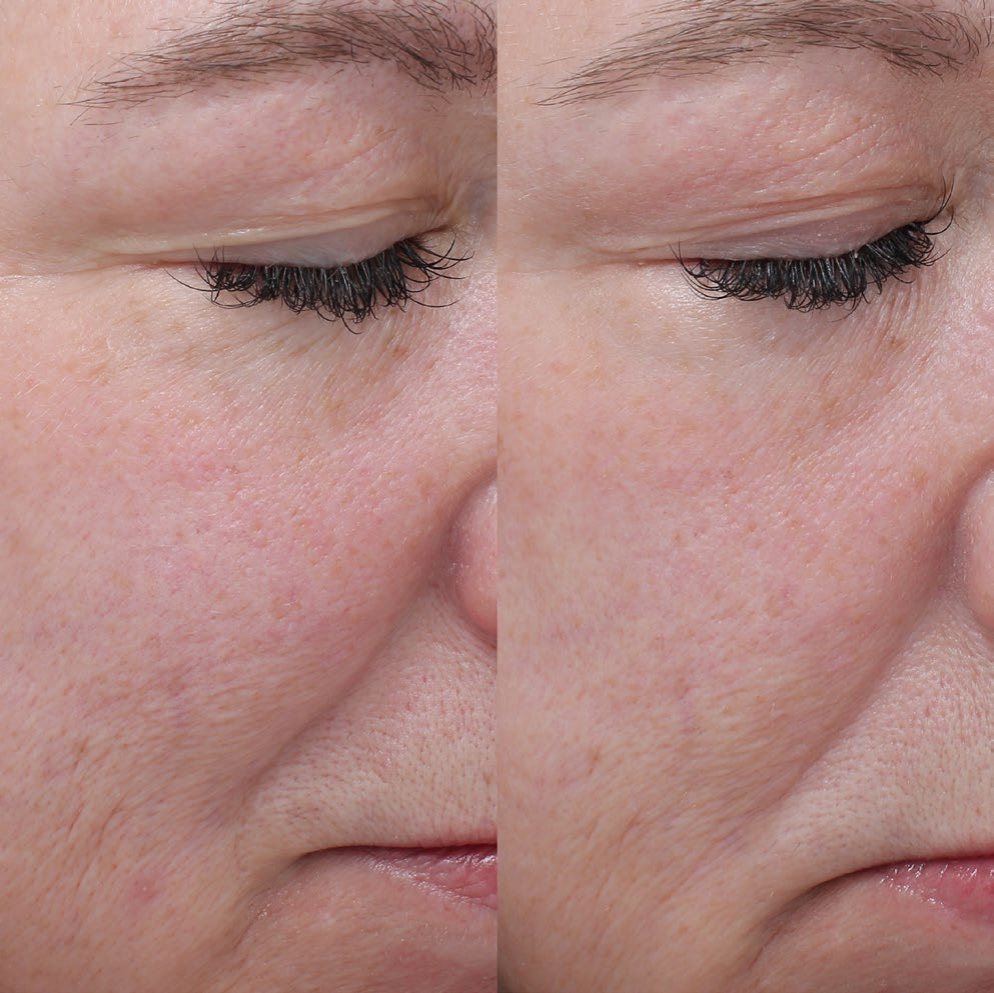
Patient 1. This is a 52‐year‐old female subject from Group A at Baseline (left) and Week 12 (right).

**FIGURE 3:**
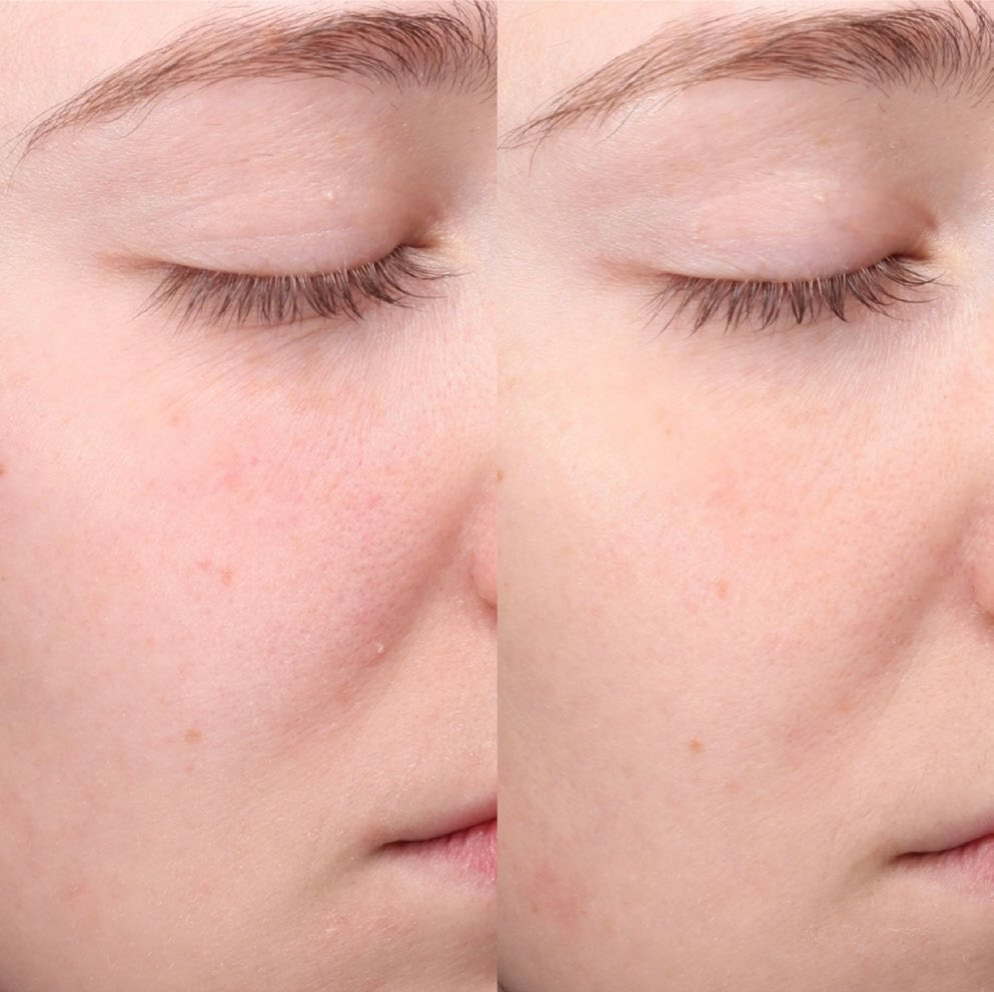
Patient 2. This is a 28‐year‐old female subject from Group A at Baseline (left) and Week 12 (right).

**FIGURE 4:**
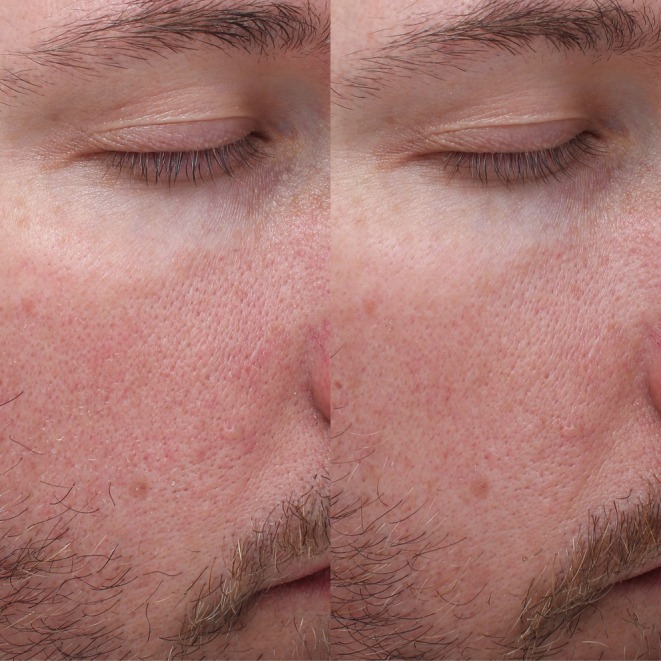
Patient 3. This is a 33‐year‐old male subject from Group B at Baseline (left) and Week 12 (right).

**FIGURE 5:**
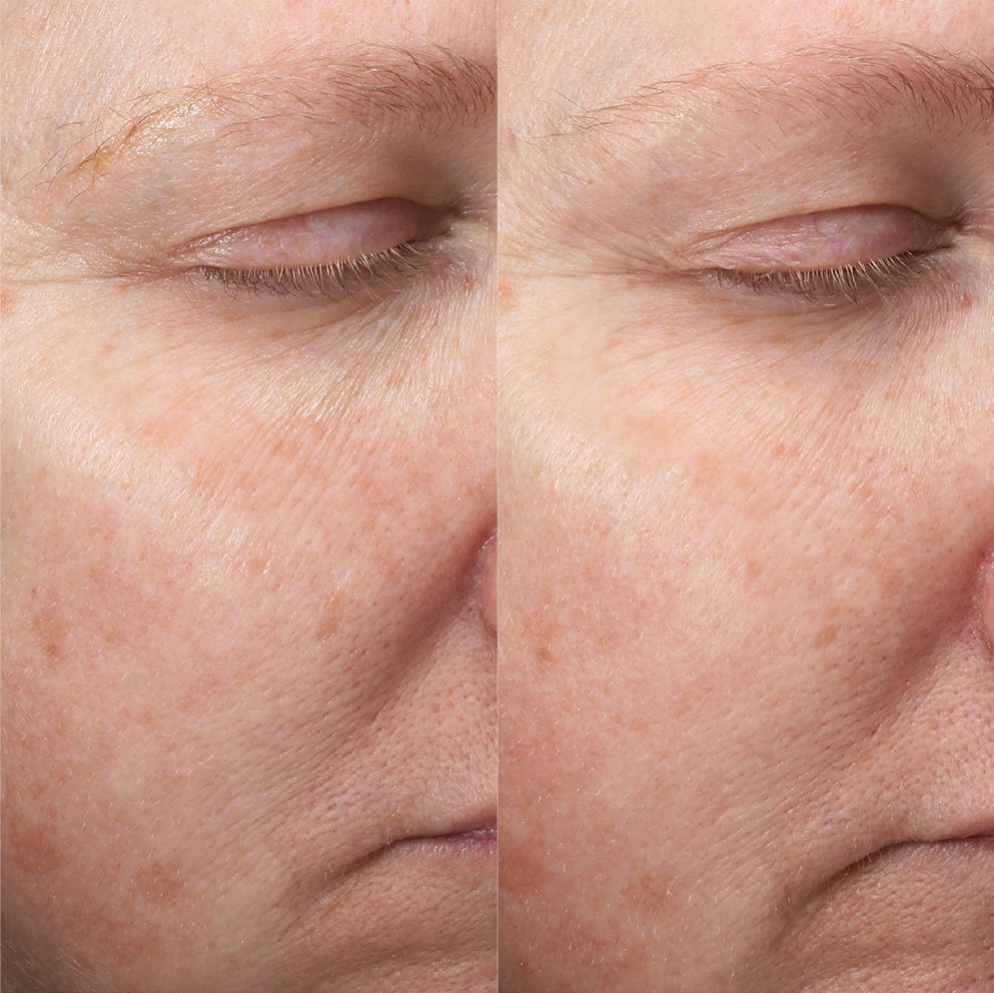
Patient 4. This is a 46‐year‐old female subject from Group B at Baseline (left) and Week 12 (right).

Repeated measures ANOVA demonstrated significant improvements across nine of eleven facial skin quality parameters. The most substantial improvements included oiliness, which showed a 75% reduction in severity scores from baseline to Week 12 (*p* < 0.01), acne scarring which improved by 62% (*p* < 0.01), dryness by 34% (*p* < 0.01), and fine lines by 20% (*p* < 0.01). Significant improvements were observed in pigmentation (27%, *p* < 0.01), roughness (28%, *p <* 0.01), wrinkles (23%, *p* < 0.05), redness (18%, *p* < 0.05), and crepiness (35%, *p* < 0.01). Only improvements in laxity and pore size failed to achieve statistical significance. Pre‐ and post‐treatment images showing facial skin quality improvement in male and female subjects from both groups are shown in Figures 2, 3, 4, 5.

### 3.4 Investigator Assessment of Facial Skin Quality Markers

Investigator assessment of improvements in the twelve Investigator Clinical Grading assessments increased over time across nearly all skin quality parameters (Table 7). By Week 12, improvement was highest for glow/luminosity (91.5%), dullness (89.6%), and skin tone (89.6%), indicating strong and consistent visible improvements in skin brightness and color uniformity. Improvement also occurred for hydration (83.3%), laxity (81.5%), and dryness (80.8%), suggesting clear evidence of enhanced moisture and skin firmness. For fine lines, pore size, and redness, improvement ranged from 70.5% to 78.6%, showing moderate but consistent recognition of improvement. The lowest improvement was reported for acne scars, with only 69.6% improved by Week 12.

**TABLE 7:** Investigator assessed improvements in facial skin quality markers.

Skin quality marker, *n* (%)	Week 4	Week 8	Week 12	Significance
Glow/Luminosity	39 (81.2)	44 (91.7)	43 (91.5)	*p* < 0.01
Dullness	38 (79.2)	44 (91.7)	43 (89.6)	*p* < 0.01
Skin tone	34 (70.8)	41 (85.4)	43 (89.6)	*p* < 0.05
Dryness	22 (84.6)	20 (76.9)	21 (80.8)	*p* = NS
Oiliness	10 (88.7)	15 (86.7)	13 (86.7)	*p* = NS
Hydration	37 (77.1)	36 (75.0)	40 (83.3)	*p* < 0.01
Laxity	19 (70.4)	20 (74.1)	22 (81.5)	*p* = NS
Fine lines	27 (64.3)	34 (81.0)	33 (78.6)	*p* < 0.05
Hyperpigmentation	32 (69.6)	30 (65.2)	39 (84.8)	*p* < 0.01
Pore size	26 (56.5)	33 (71.7)	35 (76.1)	*p* = NS
Redness	26 (59.1)	32 (72.7)	31 (70.5)	*p* = NS
Acne scars	9 (39.1)	15 (65.2)	16 (69.6)	*p* = NS

*Note:* Subjects with a baseline score of none for each marker were excluded.

Abbreviation: NS, not significant.

Using Cochran's *Q* test, significant increases in improvement over time were observed for glow/luminosity (*p* < 0.01), skin tone (*p* < 0.05), and hydration (*p* < 0.01). Pairwise comparisons using McNemar's test showed that most markers achieved significant improvement from baseline to Week 12, including dullness (*p* < 0.01), fine lines (*p* < 0.05), and pigmentation (*p* < 0.01). The progressive and significant increases in improvement support the clinical skin improvements observed throughout the study period.

### 3.5 Investigator Improvement in Overall Neck Skin Quality

Across all parameters, the mean scores improved between baseline and Week 12, suggesting a visible reduction in perceived severity over time by investigators with some variation in the rate and magnitude of improvement between groups. Both groups demonstrated gradual reductions in mean severity scores across most parameters (Table 8), with lower mean ratings indicating reduced severity. The largest overall improvements were observed in roughness (1.47 to 0.86), pigmentation (1.53 to 1.00), and fine lines (1.67 to 1.13). Redness dropped sharply by Week 8 (0.92) and increased by Week 12 (1.08), while remaining improved over baseline (1.62). Repeated measures ANOVA confirmed significant improvements across all eight neck skin quality parameters. The most pronounced improvements were roughness, which decreased by 41% from baseline to Week 12 (*p* < 0.01), and pigmentation, which improved by 35% (*p* < 0.01). Fine lines showed a 32% improvement in severity scores (*p* < 0.01), while wrinkles decreased by 16% (*p* < 0.05). Additional significant improvements included laxity (29%, *p* < 0.01), crepiness (26%, *p <* 0.01), uneven skin tone (35%, *p <* 0.01), and redness (33%, *p <* 0.05).

**TABLE 8:** Investigator ratings of improvements in neck skin quality.

Skin quality marker, *n* (%)	Baseline	Week 4	Week 8	Week 12	Significance
Wrinkles	1.75	1.56	1.5	1.47	*p* < 0.05
Laxity	1.89	1.41	1.54	1.34	*p* < 0.01
Crepiness	1.81	1.38	1.59	1.34	*p* < 0.01
Fine lines	1.67	1.41	1.26	1.13	*p* < 0.01
Redness	1.62	1.42	0.92	1.08	*p* < 0.05
Uneven skin tone	1.54	1.26	1.11	1.0	*p* < 0.01
Hyperpigmentation	1.53	1.27	1.06	1.0	*p* < 0.01
Roughness	1.47	1.06	0.97	0.86	*p* < 0.01

*Note:* Excludes subjects with a value of 0 at baseline.

### 3.6 Investigator Assessment of Neck Skin Quality Markers

Investigator‐assessment of improvements in neck skin quality markers showed consistent and progressive increases over the 12‐week study (Table 9). By Week 12, the highest level of improvement was observed for smoothness (94.4%), firmness (88.6%), and fine lines (87.2%), suggesting a strong visible response to treatment in structural and textural domains. Similarly, hyperpigmentation (87.5%) and skin tone (81.2%) showed substantial improvement, indicating enhanced pigmentation uniformity and brightness. Wrinkle improvement was initially high at Week 4 (72.2%), then decreasing at Week 8 (55.6%), before increasing again to 80.6% by Week 12 (80.6%). Redness showed the lowest improvement at Week 4 (53.8%) and Week 8 (57.7%) but improved notably by Week 12 (80.8%).

**TABLE 9:** Investigator assessed improvements in neck skin quality markers.

Skin quality marker, *n* (%)	Week 4	Week 8	Week 12	Significance
Smoothness	27 (75.0)	30 (83.3)	34 (94.4)	*p* < 0.01
Firmness	29 (82.9)	27 (77.1)	31 (88.6)	*p* < 0.01
Fine lines	26 (66.7)	31 (79.5)	34 (87.2)	*p* < 0.01
Hyperpigmentation	19 (59.4)	23 (71.9)	28 (87.5)	*p* < 0.01
Skin tone	30 (62.5)	35 (72.9)	39 (81.2)	*p* < 0.01
Wrinkles	26 (72.2)	20 (55.6)	29 (80.6)	*p* < 0.05
Redness	14 (53.8)	15 (57.7)	21 (80.8)	*p* = NS

*Note:* Subjects with a baseline score of none for each marker were excluded.

Abbreviation: NS, not significant.

Statistical evaluation using Cochran's *Q* test confirmed significant improvements in investigator assessments for all neck skin quality markers. The strongest statistical evidence for increasing improvement over time was observed for smoothness (*p* < 0.01), firmness (*p* < 0.01), and fine lines (*p* < 0.01). McNemar's test pairwise comparisons revealed significant increases in improvement from baseline to Week 12 for hyperpigmentation (*p* < 0.01), skin tone (*p* < 0.01), and wrinkles (*p* < 0.05). The progressive and significant increases in improvement support the clinical relevance of neck skin improvements observed throughout the study period.

### 3.7 Investigator‐Rated Product Tolerability

Overall tolerability across the 12‐week period was favorable, and most reported treatment‐related adverse events were mild and infrequent. Among all subjects, erythema was the most common reaction, followed by dryness, scaling, and edema.

### 3.8 Subject Satisfaction

At the baseline visit, most enrolled subjects indicated they had a number of facial skin concerns (Table 10); however, subject satisfaction with their treatment results was high at the final study visit (Table 11) with substantial improvements in skin appearance and self‐confidence. The percentage of subjects agreeing that their skin looked and felt healthier was 96%, and perceived youthfulness was 94%. Confidence and perception of overall improvement were each reported by 83% and 88% of subjects. When assessed by groups, there were no significant between‐group differences. Among all subjects, 81.2% indicated they would continue using the product and 91.7% stated they would recommend it to others.

**TABLE 10:** Baseline subject facial skin concerns.

Quality marker, *n* (%)	All (*N* = 48)	Group A (*n* = 24)	Group B (*n* = 24)
Redness	25 (52.1)	10 (41.7)	15 (62.5)
Hyperpigmentation	19 (39.6)	11 (45.8)	8 (33.3)
Uneven skin tone	30 (62.5)	14 (58.3)	16 (66.7)
Scars (Acne)	19 (39.6)	11 (45.8)	8 (33.3)
Pore size	26 (54.2)	15 (62.5)	11 (45.8)
Dullness	15 (31.2)	9 (37.5)	6 (25.0)
Laxity	9 (18.8)	6 (25.0)	3 (12.5)
Crepiness	3 (6.3)	1 (4.2)	2 (8.3)
Dryness	23 (47.9)	12 (50.0)	11 (45.8)
Lack of luminosity	20 (41.7)	9 (37.5)	11 (45.8)
Rough/uneven texture	17 (35.4)	8 (33.3)	9 (37.5)
Fine lines	27 (56.2)	11 (45.8)	16 (66.7)
Wrinkles	23 (47.9)	13 (54.2)	10 (41.7)

*Note:* Only the percentages of subjects who responded “Yes” for each facial skin concern are shown.

**TABLE 11:** Subject satisfaction with treatment results.

	Week 12
Skin looks and feels healthier	96%
Look young/more youthful	94%
Notice overall improvement	88%
Feel more confident about skin	83%

## 4 Discussion

The objective of this study was to assess changes in skin quality from an anti‐aging 10‐peptide serum designed to improve skin quality markers in facial and neck skin in two subject groups: those not currently using prescription, medical grade, advanced or physician‐dispensed skincare products and those who were currently using prescription, medical grade, advanced or physician‐dispensed skincare products. At baseline, all subjects were rated by the Investigators as having issues based on a range of skin quality markers. Following twice‐daily application for 12 weeks, there were significant progressive improvements in most Investigator ratings of facial and neck skin quality parameters, which were similar in both treatment groups.

The greatest overall Week 12 facial skin improvements were luminosity, dullness, skin tone, and dryness while significant overall neck skin improvements were wrinkles, laxity, and crepiness. Investigator assessments for important facial skin quality improvements such as luminosity, dullness, and skin tone were also significant as were neck skin qualities of smoothness, fine lines, and crepiness.

Importantly, subject satisfaction ratings for their treatment results were high. As a result, subjects reported substantial improvements in a more healthy, youthful appearance of their skin which were associated with greater self‐confidence for both treatment groups. The majority of subjects indicated they would continue to use the anti‐aging 10‐peptide serum and would recommend it to friends.

It is noteworthy that all the observed improvements in skin quality markers assessed in this study were incremental to any improvements achieved by prior use of OTC and professional‐grade skin treatments.

These results are in agreement with an unpublished 8‐week, multicenter study that assessed improvements in skin quality and subject satisfaction after using this anti‐aging 10‐peptide serum [38]. Among the treated subjects (*N* = 158), investigators reported significant improvement in overall skin health (*p* < 0.01) and skin hydration (*p* < 0.01) while nearly all subjects saw an improvement in their overall skin health (91%) and skin hydration (85%). Most subjects (94%) reported their skin felt more rejuvenated as early as Week 4.

The anti‐aging 10‐peptide serum described in this study has several advantages for facial rejuvenation. It is well‐tolerated and compatible with other over‐the‐counter and professional skincare products. In addition, the presence of antioxidants protects against damaging environmental insults, such as ultraviolet and visible light and air pollution.

Limitations of the present study include an open‐label design and the lack of a placebo group which might have further demonstrated the beneficial effects of the anti‐aging 10‐peptide serum. It is known that sunscreen use contributes to improvements in skin health, and its role could be considered when interpreting study outcomes. In this study, subjects followed sunscreen application and reapplication consistent with FDA labeling and Skin Cancer Foundation recommendations, reflecting standard‐of‐care practice; while supportive of skin health, these measures represent routine background care within the overall study design rather than an isolated contributor to outcomes. A study of longer duration might have demonstrated progressive improvements in skin quality parameters.

## 5 Conclusion

The twice‐daily application of a novel anti‐aging 10‐peptide serum restored numerous markers of facial and neck skin quality that were incremental to both groups. Statistically significant improvements were seen in all subjects, including those with and without a prior existing skin care regimen in terms of their Global Improvements as well as most of the key markers associated with skin quality. Skin quality improvements included enhanced firmness, elasticity, smoothness, and radiance, accompanied by reduced self‐consciousness and increased self‐confidence. These improvements in skin quality are generally associated with decreased feelings of self‐consciousness and an improvement in self‐confidence. Importantly, the serum aligns with the increasing interest for advanced, scientifically validated formulations that deliver measurable improvements in overall skin quality and support the pursuit of smoother, more radiant skin. The product is well‐tolerated and compatible with concomitant over‐the‐counter and professional skincare products. These results underscore the serum's ability to address the growing consumer demand for scientifically validated, multi‐functional formulations that deliver measurable enhancements in overall skin quality and contribute to a more flawless appearance.

## Author Contributions

The authors shared equally in the design and execution of this study and the preparation of this manuscript.

## Ethics Statement

The authors confirm that the ethical policies of the journal, as noted on the journal's author guidelines page, have been adhered to and the appropriate ethical review committee approval has been received.

## Conflicts of Interest

The authors declare no conflicts of interest.

## Data Availability

The data that support the findings of this study are available from the corresponding author upon reasonable request.

## References

[1] Z. Al Timimi , A. F. Al‐Rubaye , and D. M. Diwan , “A Comprehensive Study of Laser Use in Dermatology: Assessing the Safety, Innovations, and Effectiveness of Laser Technology for Skin Treatment,” Irish Journal of Medical Science 194 (2025): 923–932. 40138057 10.1007/s11845-025-03942-3

[2] J. L. Cohen , G. J. Goodman , A. T. De Almeida , et al., “Decades of Beauty: Achieving Aesthetic Goals Throughout the Lifespan,” Journal of Cosmetic Dermatology 22 (2023): 2889–2901. 37632289 10.1111/jocd.15968

[3] S. F. Weiner , “Radiofrequency Microneedling: Overview of Technology, Advantages, Differences in Devices, Studies, and Indications,” Facial Plastic Surgery Clinics of North America 27 (2019): 291–303. 31280844 10.1016/j.fsc.2019.03.002

[4] K. W. Broder and S. R. Cohen , “An Overview of Permanent and Semipermanent Fillers,” Plastic and Reconstructive Surgery 118, no. 3 Suppl (2006): 7S–14S. 10.1097/01.prs.0000234900.26676.0b 16936539

[5] K. Goldie , M. Kerscher , S. G. Fabi , et al., “Skin Quality—A Holistic 360° View: Consensus Results,” Clinical, Cosmetic and Investigational Dermatology 14 (2021): 643–654. 34163203 10.2147/CCID.S309374 PMC8214518

[6] C. Martschin , R. Bahhady , J. Li , et al., “Development and Validation of a Novel Holistic Skin Quality Assessment Scale,” Journal of Cosmetic Dermatology 24 (2025): e16615. 39382191 10.1111/jocd.16615 PMC11743243

[7] S. Humphrey , S. Manson Brown , S. J. Cross , and R. Mehta , “Defining Skin Quality: Clinical Relevance, Terminology, and Assessment,” Dermatologic Surgery 47 (2021): 974–981. 34148998 10.1097/DSS.0000000000003079 PMC8231670

[8] B. Fink and N. Neave , “The Biology of Facial Beauty,” International Journal of Cosmetic Science 27 (2005): 317–325. 18492169 10.1111/j.1467-2494.2005.00286.x

[9] H. E. van den Elzen , A. J. Barends , M. van Vugt , et al., “Facial Aesthetic Minimally Invasive Procedure: More Than Just Vanity, a Social‐Psychological Approach,” Journal of Cosmetic Dermatology 22 (2023): 2063–2070. 36852750 10.1111/jocd.15678

[10] A. Morita , “Tobacco Smoke Causes Premature Skin Aging,” Journal of Dermatological Science 48 (2007): 169–175. 17951030 10.1016/j.jdermsci.2007.06.015

[11] T. Yazdanparast , H. Hassanzadeh , S. A. Nasrollahi , et al., “Cigarettes Smoking and Skin: A Comparison Study of the Biophysical Properties of Skin in Smokers and Non‐Smokers,” Tanaffos 18 (2019): 163–168. 32440305 PMC7230126

[12] K. A. Merin , M. Shaji , and R. Kameswaran , “A Review on Sun Exposure and Skin Diseases,” Indian Journal of Dermatology 67 (2022): 625. 10.4103/ijd.ijd_1092_20 PMC9971785 36865856

[13] F. Flament , R. Bazin , S. Laquieze , V. Rubert , E. Simonpietri , and B. Piot , “Effect of the Sun on Visible Clinical Signs of Aging in Caucasian Skin,” Clinical, Cosmetic and Investigational Dermatology 6 (2013): 221–232. 24101874 10.2147/CCID.S44686 PMC3790843

[14] A. Jakubczyk‐Słabicka , T. Kostrzewa , W. Barańska‐Rybak , and M. Górska‐Ponikowska , “Skin and Scalp Under Exposure to High‐Energy Visible Light: The Current Perspective,” Archives of Dermatological Research 317 (2025): 521. 40042632 10.1007/s00403-025-04021-4 PMC11882610

[15] C. H. Huang , S. C. Chen , Y. C. Wang , C. F. Wang , C. H. Hung , and S. S. Lee , “Detrimental Correlation Between Air Pollution With Skin Aging in Taiwan Population,” Medicine (Baltimore) 101 (2022): e29380. 35945750 10.1097/MD.0000000000029380 PMC9351911

[16] J. Krutmann , T. Schikowski , A. Morita , and M. Berneburg , “Environmentally‐Induced (Extrinsic) Skin Aging: Exposomal Factors and Underlying Mechanisms,” Journal of Investigative Dermatology 141 (2021): 1096–1103. 33541724 10.1016/j.jid.2020.12.011

[17] A. Vierkötter , T. Schikowski , U. Ranft , et al., “Airborne Particle Exposure and Extrinsic Skin Aging,” Journal of Investigative Dermatology 130 (2010): 2719–2726. 20664556 10.1038/jid.2010.204

[18] J. J. McArdle , T. L. Lentz , V. Witzemann , H. Schwarz , S. A. Weinstein , and J. J. Schmidt , “Waglerin‐1 Selectively Blocks the Epsilon Form of the Muscle Nicotinic Acetylcholine Receptor,” Journal of Pharmacology and Experimental Therapeutics 289 (1999): 543–550. 10087048

[19] A. D. Widgerow , L. I. Jiang , and A. Calame , “A Single‐Center Clinical Trial to Evaluate the Efficacy of a Tripeptide/Hexapeptide Antiaging Regimen,” Journal of Cosmetic Dermatology 18 (2019): 176–182. 29504212 10.1111/jocd.12507

[20] F. Gorouhi and H. I. Maibach , “Role of Topical Peptides in Preventing or Treating Aged Skin,” International Journal of Cosmetic Science 31 (2009): 327–345. 19570099 10.1111/j.1468-2494.2009.00490.x

[21] N. Hajem , A. Chapelle , J. Bignon , et al., “The Regulatory Role of the Tetrapeptide AcSDKP in Skin and Hair Physiology and the Prevention of Ageing Effects in These Tissues—A Potential Cosmetic Role,” International Journal of Cosmetic Science 35 (2013): 286–298. 23488645 10.1111/ics.12046

[22] V. Raikou , A. Varvaresou , I. Panderi , and E. Papageorgiou , “The Efficacy Study of the Combination of Tripeptide‐10‐Citrulline and Acetyl Hexapeptide‐3. A Prospective, Randomized Controlled Study,” Journal of Cosmetic Dermatology 16 (2017): 271–278. 28150423 10.1111/jocd.12314

[23] J. Kim , S. Kang , H. Kwon , H. Moon , and M. C. Park , “Dual Functional Bioactive‐Peptide, AIMP1‐Derived Peptide (AdP), for Anti‐Aging,” Journal of Cosmetic Dermatology 18 (2019): 251–257. 29921010 10.1111/jocd.12671

[24] N. S. Trookman , R. L. Rizer , R. Ford , E. Ho , and V. Gotz , “Immediate and Long‐Term Clinical Benefits of a Topical Treatment for Facial Lines and Wrinkles,” Journal of Clinical and Aesthetic Dermatology 2 (2009): 38–43. PMC2923951 20729942

[25] S. Pitman , “Tripeptide Features as Key Ingredient in Anti‐Aging Launches,” (2024), CosmeticsDesign.com, accessed October 21, 2025, https://www.cosmeticsdesign.com/Article/2007/04/16/tripeptide‐features‐as‐key‐ingredient‐in‐anti‐aging‐launches/.

[26] K. Sivaraman and C. Shanthi , “Matrikines for Therapeutic and Biomedical Applications,” Life Sciences 214 (2018): 22–33. 30449450 10.1016/j.lfs.2018.10.056

[27] S. J. Lim , D. J. Min , S. Kim , et al., “Pseudoalteromone A, a Ubiquinone Derivative From Marine *Pseudoalteromonas* spp., Suppresses Melanogenesis,” Marine Drugs 19 (2021): 612. 34822483 10.3390/md19110612 PMC8618130

[28] A. Novitasari , E. Rohmawaty , and A. M. Rosdianto , “ *Physalis angulata* Linn. as a Medicinal Plant (Review),” Biomedical Reports 20 (2024): 47. 38357237 10.3892/br.2024.1735 PMC10865294

[29] Y. Y. Sung , M. Kim , D. S. Kim , and E. Son , “ *Glycine soja* Leaf and Stem Extract Ameliorates Atopic Dermatitis‐Like Skin Inflammation by Inhibiting JAK/STAT Signaling,” International Journal of Molecular Sciences 26 (2025): 4560. 40429704 10.3390/ijms26104560 PMC12110808

[30] H. Fu , S. You , D. Zhao , et al., “Tremella Fuciformis Polysaccharides Inhibit UVA‐Induced Photodamage of Human Dermal Fibroblast Cells by Activating Up‐Regulating Nrf2/Keap1 Pathways,” Journal of Cosmetic Dermatology 20 (2021): 4052–4405. 33686752 10.1111/jocd.14051

[31] J. H. Chiang , F. J. Tsai , T. H. Lin , J. S. Yang , and Y. J. Chiu , “ *Tremella fuciformis* Inhibits Melanogenesis in B16F10 Cells and Promotes Migration of Human Fibroblasts and Keratinocytes,” In Vivo 36 (2022): 713–722. 35241526 10.21873/invivo.12757 PMC8931924

[32] H. Cho , J. Yang , J. Y. Kang , and K. E. Kim , “Inhibitory Effects of Fermented Sprouted Oat Extracts on Oxidative Stress and Melanin Overproduction,” Antioxidants 13 (2024): 544. 38790649 10.3390/antiox13050544 PMC11117960

[33] H. S. Kim , H. J. Hwang , W. D. Seo , and S. H. Do , “Oat (*Avena sativa* L.) Sprouts Restore Skin Barrier Function by Modulating the Expression of the Epidermal Differentiation Complex in Models of Skin Irritation,” International Journal of Molecular Sciences 24 (2023): 17274. 38139104 10.3390/ijms242417274 PMC10743458

[34] M. Boen , M. Alhaddad , D. C. Wu , and M. P. Goldman , “A Prospective Double‐Blind, Placebo‐Controlled Clinical Trial Evaluating the Efficacy of a Novel Combination of Hyaluronic Acid Serum and Antioxidant Cream for Rejuvenation of the Aging Neck,” Journal of Clinical and Aesthetic Dermatology 13 (2020): 13–18. PMC7716741 33282096

[35] S. G. Fabi , L. Cattelan , B. Caughlin , J. S. Pérez , M. Cirrincione , and S. Dayan , “Unlocking the Psycho‐Social‐Dermal Axis: A Double Blinded Randomized Placebo Controlled Study Unveiling the Influence of a Novel Topical Formulation on Skin Quality, Attractiveness, Quality of Life, and Sexual Satisfaction,” Journal of Cosmetic Dermatology 23 (2024): 2905–2917. 39073288 10.1111/jocd.16496

[36] A. Chiu , J. R. Montes , G. Munavalli , A. Shamban , S. Chawla , and S. Abrams , “Improved Patient Satisfaction With Skin After Treatment of Cheek Skin Roughness and Fine Lines With VYC‐12L: Participant‐Reported Outcomes From a Prospective, Randomized Study,” Aesthetic Surgery Journal 43 (2023): 1367–1375. 37074002 10.1093/asj/sjad111 PMC10575618

[37] L. Lin , X. Chen , C. Liu , et al., “Comparative Efficacy of Topical Interventions for Facial Photoaging: A Network Meta‐Analysis,” Scientific Reports 15 (2025): 26889. 40707570 10.1038/s41598-025-12597-0 PMC12289910

[38] Colorescience , Pep Up Collagen Renewal. Face & Neck Treatment Multicenter Study. Unpublished Data on File (Colorescience Inc, 2017).

